# Microscopic Preparations

**Published:** 1878-06

**Authors:** 


### MICROSCOPIC PREPARATIONS.

While many minute objects need but little skill to exhibit them under the microscope, it is nevertheless true that structural details require the expenditure of much time and labor. No one can become a microscopist in a day, and the study of many years will only bring us to the threshold of Nature's temple. These remarks are suggested by a criticism upon some of the objects displayed at the annual meeting of the Academy of Natural Sciences in Philadelphia. The committee of the biological and microscopical section of the Academy say in their report, in which full justice is done to the beauty and skill displayed in the preparations: "It would be desirable in such work, if other parts than the vessels, such as the connective tissues, could be properly stained and demonstrated at the same time. This would involve some loss of transparency, but the educational value would be much increased. No preparation is well enough shown unless all its tissues are demonstrated." These remarks apply with special force to a large number of the specimens prepared and kept for sale by professional opticians. Many of them are beautiful objects, and well adapted for display, but are of little histological use. If the microscope is to be anything more than an expensive toy, it behooves the student to be indefatigable in personal work. A true knowledge of minute structure can only be acquired by experiment with a variety of methods of examination and differentiation and presentation. It is doubtful if any single specimen can demonstrate all the tissues satisfactorily.

DIATOMS.

The variety and beauty of diatoms cause them to be great favorites with microscopists. Every part of the globe is ransacked to supply our cabinets, and there is some danger of a diatom-mania, which shall turn aside some workers from the true investigation of structure, and render them mere collectors and namers of specimens. The life history of these minute algæ is of far greater importance than their classification, and will elucidate many biological problems. It is desirable that those who have the opportunity should study and report the phenomena of their growth and propagation. Although much has been done in this direction, the subject is still a fertile one.

Art is now able to draw lines with a diamond upon glass of most wonderful fineness; so much so, that Nobert is able to execute those of such delicacy that it would require over one hundred thousand, with their interspaces, to occupy an inch. Mr. Webb, of London—and we have one of his specimens—engraves the Lord's prayer upon glass, with its two hundred and twenty-seven letters, so as to occupy only the one twenty thousand seven hundred and twentieth of a square inch ($\frac{1}{20720}$), or, upon a space which, if the whole Bible, Old and New Testament, with its four million seven hundred and three thousand four hundred and forty letters, was engraved with the same fineness, it could be put upon less than an inch square. Yet these seemingly exquisite fine lines appear coarse and jagged under a fine lens, while the lines upon a diatom called *amphipleura pellucida* exhibit perfect smoothness, as do the lines on all diatoms.

"When a microscopist wishes to test a lens to ascertain whether its performance is fully up to the standard which a good glass of its power, or degree of magnification, should reach, he selects from his cabinet a glass slide mounted, as it is termed, with diatoms having lines of certain fineness which, as is generally agreed upon, a lens of its power should be able to resolve and show clearly. If it does, he regards it as a good one; if it does not, he considers it inferior. Right here we will mention that resolution and magnification do not, as might be supposed by some, go hand in hand. A lens may magnify a desired number of diameters, and yet not possess sufficient fineness to bring into view surface markings that one of much less power would readily do. Moller, of Germany, mounts upon glass slides twenty diatoms in a line having "markings" ranging in fineness from three or four to the one-thousandth of an inch to over a hundred to one-thousandth of an inch. The *pleurosigma angulatum*, a diatom more used than any other among microscopists as a general test for medium powers, is the eleventh on the scale. It averages about fifty markings to the one-thousandth of an inch. A lens of a quarter inch focal length, magnifying about two hundred diameters, of medium quality, will show these lines without difficulty. A fine one of considerable less power, a half inch in focal length, magnifying one hundred diameters, will also do it. The next on the scale is *grammatophora subtilissima*, the lines of which, on an average, in comparing many of these diatoms, are over sixty to the one-thousandth of an inch, but there are some having over eighty, according to some measurements reported. *Surirella gemma* has two sets of lines, differing very much in fineness. The finest, the longitudinal, average over eighty to the thousandth of an inch. A lens of a focal length of a quarter inch, fifth, sixth, or eighth, especially if it is what is termed a dry one, that exhibits well the longitudinal lines, may be safely regarded as a very good one, and its performance in investigations in any department of science will be entirely satisfactory. Besides the test diatoms we have mentioned, there are many others, as the *pleurosigma formosum*, *p. strigile*, *p. Balticum*, *p. hippocampus*, *p. quadratum*, *p. fasciola*, *p. macrum*, *navicula rhomboides*, *nitzchia sigmoidea*, *hyalodiscus subtilis*, etc, There are some met with that there are reasons to believe possess markings of such fineness, that no lens has yet been made, notwithstanding the great degree of perfection to which their performance has been brought, that can resolve them."

